# Separability of Acute Cerebral Infarction Lesions in CT Based Radiomics: Toward Artificial Intelligence-Assisted Diagnosis

**DOI:** 10.1155/2020/8864756

**Published:** 2020-11-15

**Authors:** Yun Guan, Peng Wang, Qi Wang, Peihao Li, Jianchao Zeng, Pinle Qin, Yanfeng Meng

**Affiliations:** ^1^North University of China-Taiyuan Central Hospital Joint Innovation Institute, 3 Xueyuan Road, Taiyuan, Shanxi 030051, China; ^2^College of Big Data, North University of China, 3 Xueyuan Road, Taiyuan, Shanxi 030051, China; ^3^Taiyuan Central Hospital of Shanxi Medical University, 5 Dong San Dao Lane, Jiefang Street, Taiyuan, Shanxi 030009, China; ^4^School of Information and Communication Engineering, North University of China, 3 Xueyuan Road, Taiyuan, Shanxi 030051, China

## Abstract

This study aims at analyzing the separability of acute cerebral infarction lesions which were invisible in CT. 38 patients, who were diagnosed with acute cerebral infarction and performed both CT and MRI, and 18 patients, who had no positive finding in either CT or MRI, were enrolled. Comparative studies were performed on lesion and symmetrical regions, normal brain and symmetrical regions, lesion, and normal brain regions. MRI was reconstructed and affine transformed to obtain accurate lesion position of CT. Radiomic features and information gain were introduced to capture efficient features. Finally, 10 classifiers were established with selected features to evaluate the effectiveness of analysis. 1301 radiomic features were extracted from candidate regions after registration. For lesion and their symmetrical regions, there were 280 features with information gain greater than 0.1 and 2 features with information gain greater than 0.3. The average classification accuracy was 0.6467, and the best classification accuracy was 0.7748. For normal brain and their symmetrical regions, there were 176 features with information gain greater than 0.1, 1 feature with information gain greater than 0.2. The average classification accuracy was 0.5414, and the best classification accuracy was 0.6782. For normal brain and lesions, there were 501 features with information gain greater than 0.1 and 1 feature with information gain greater than 0.5. The average classification accuracy was 0.7480, and the best classification accuracy was 0.8694. In conclusion, the study captured significant features correlated with acute cerebral infarction and confirmed the separability of acute lesions in CT, which established foundation for further artificial intelligence-assisted CT diagnosis.

## 1. Introduction

Globally, stroke is still the leading cause of mortality and disability, and there are substantial economic costs for poststroke care [1–4]. In practice, CT is the preferred radiologic modality for patients with stroke-like clinical manifestation, since it is immediately available, cost effective, and capable of differentiating brain disorders [5]. CT is very sensitive in detecting intracranial hemorrhagic stroke and chronic ischemic stroke. CT detects acute cerebral infarction (ACI) in terms of decrease of CT attenuation, loss of gray-white matter differentiation, sulcal effacement, and other indirect signs [6]. In practice, radiologists often encounter poor accuracy in diagnosing acute infarct by CT, with accuracy rate ≤67% within 3 hours [5].

Patient, who has stroke-like symptom but CT showed negative findings, needs MRI [7]. MR diffusion-weighted imaging (DWI) can detect ischemic lesions within minutes of symptoms, which is an extremely sensitive technology and used for estimating whether thrombolysis is appropriate. However, MRI is still not the primary modality, because it is time-consuming, which may lead to miss time window of thrombolysis, costing expensive, and various contradictions [8, 9]. Studies demonstrated that better clinical outcomes correlated with earlier diagnosis of ischemic stroke [10]. Therefore, it is essential to improve the accuracy rate of early recognizing ACI by CT within the time window.

In this study, we hypothesize that ACI lesions in CT are separable by combining image registration, accurate lesion location, radiomic feature extraction, and information gain calculation, so as to establish a foundation for artificial intelligence-assisted CT diagnosis.

## 2. Materials and Methods

### 2.1. Patients

This retrospective study protocol was reviewed and approved by the institutional review board of our hospital. Written informed consent was waived.

**Figure 1:**
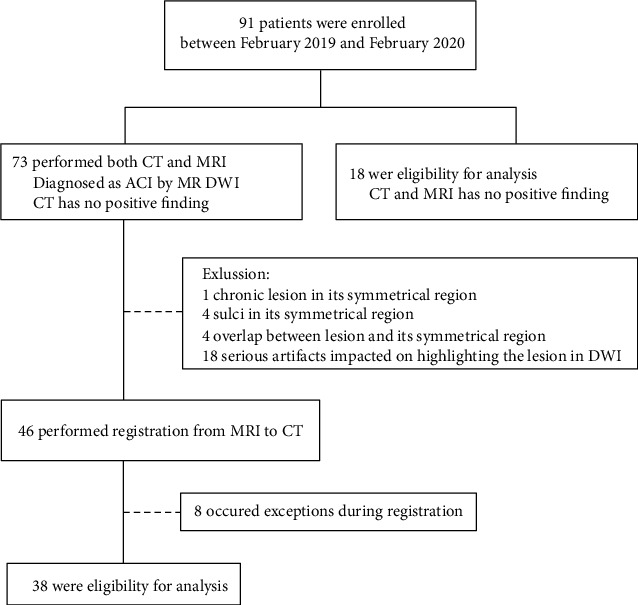
Flowchart of the recruitment produce for this study. ACI denotes acute cerebral infarction.

Between February 2019 and February 2020, we retrospectively studied 38 patients, who have performed both CT and MRI and diagnosed as ACI by DWI; meanwhile, CT has no positive finding by radiologist. Another 18 patients with no positive finding in either CT or MRI were also enrolled as normal control (Figure 1).

### 2.2. CT and MRI

CT images were acquired on a 320-MDCT scanner (Aquilion ONE, Toshiba Medical System, Otawara, Japan) with the following parameters: 120 kV and 300 mA, 5-mm slice thickness, 512 × 512 matrix, and 0.6 mm collimation.

MRI was acquired on 1.5 T MR scanner (Sonata, Siemens Healthcare, Erlangen, Germany) and 3.0 T MR scanner (MAGNETOM Skyra, Siemens Healthcare, Erlangen, Germany). The parameters of 1.5 T DWI were as follows: TR/TE = = 3800ms/84ms, slicethickness = 5mm, matrix = 128 × 128, FOV = 200 × 220, *b*value = 1000. The parameters of 3.0 T DWI were as follows: TR/TE = = 4950ms/64ms, slicethickness = 5mm, matrix = 164 × 164, FOV = 220 × 220, *b*value = 1000.

### 2.3. Registration and Candidate Region Acquisition

**Figure 2:**
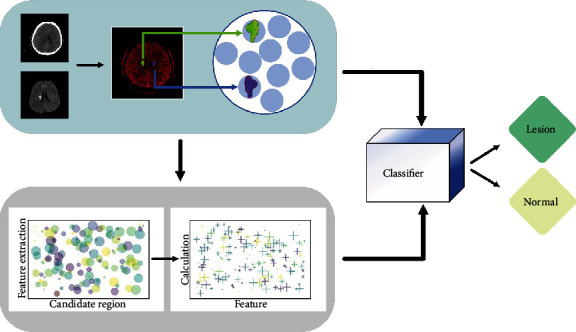
The pipeline of our methodology included three steps: registration and candidate region acquisition, feature extraction and analysis, and classifiers establishment. Firstly, CT and MRI were input to obtain lesion regions and their symmetrical regions as candidate regions through registration. Then, features were extracted and calculated from candidate regions to capture useful features for auxiliary separating acute cerebral infarction. Finally, the classifiers were introduced to separate candidate region with selected features.

**Figure 3:**
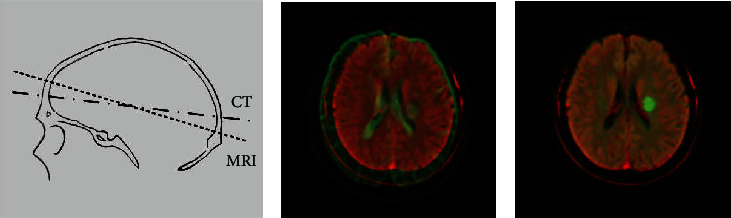
Image registration. (a) DWI was adjusted by multiple planner reconstruction to obtain a consistent angle with CT. Dotted line denoted MRI and point solid line denoted CT. (b) CT and DWI were put together to achieve coarse registration. (c) Fine registration was performed by a series of affine transformation including translation, rotation, and scaling.

The pipeline of our methodology was shown in Figure 2. Since the position and angle of CT were different from MRI for one patient, the DWI had to be registered to CT images (Figure 3). Herein, the CT images were not adjusted to avoid loss of intact information. The DWI were multiple planners reconstructed to get a consistent angle with CT and achieve coarse registration. Then, a series of affine transformations were performed to get a consistent position and achieve fine registration, including translation, rotation, and scaling transformation.

Early cerebral infarction was obvious on DWI which was sensitive to the restricted Brownian movement of water molecules in brain tissue [5]. Immediately after registration, we highlighted the lesion regions in DWI through adjusting the window width and window level. Because of CT and DWI were matched, the salient lesion position of DWI was also used as the lesion label for CT.

**Figure 4:**
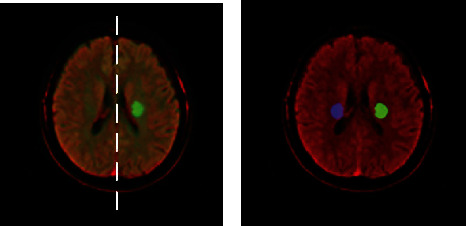
Candidate region acquisition. (a) The midline of the brain was the axis of symmetry for projecting symmetric position. (b) Depict the profile of symmetrical regions for achieving comparative analysis. The lesion regions and their symmetrical regions were served as candidate regions.

Besides delineating exact lesion regions in CT projected from DWI, we took the midline of the brain as the axis of symmetry and depicted the profile of symmetrical regions (Figure 4). Instead of simply comparing the left and right sides of the brain [11–13], the lesion regions and their symmetrical regions were served as candidate regions to reduce redundant information and achieve accurate comparative analysis.

### 2.4. Feature Extraction and Analysis

Unlike Lo et al. [13] who improved a texture feature of radiomics, our scheme was to extract features from the image firstly, and then used machine learning techniques to learn these features, so that the computer can mine the information of cerebral infarction in CT according to the acquired characteristics and then identify. Radiomic feature extraction and statistical analysis were performed to complete the plentiful features extraction and choose features which are contributing to classification, respectively.

High-throughput feature extraction was applied to search abundant information in CT images. In this study, we followed the radiomic method by Lambin et al. [14] for the extraction process, which were divided into two steps: (1) image transformation and (2) feature calculation[15]. For image transformation, feature map was constructed nonlinearly from the original images, including wavelet, square root, and logarithm. For feature calculation, the feature was calculated on the original and transformed images, including first-order statistics and gray level cooccurrence matrix.

The information gain was further introduced as a statistical standard to measure the correlation between radiomic features and ACI, which was devoted to select significative information from a great deal features of above [16–18]. Each feature was calculated out a value in terms of information gain for dichotomy, lesion, or normal regions, by the equation below:

(1) $$\begin{array}{c} H\left({\text{X}}_{i}\right)=\underset{x\in {\text{X}}_{i}}{\sum }-p\left(x\right)\log p\left(x\right), \end{array}$$

(2) $$\begin{array}{c} H\left(Y\;|\;{\text{X}}_{i}\right)=\underset{x\in {\text{X}}_{i}}{\sum }p\left(x\right)H\left(Y\;|\;{\text{X}}_{i}=x\right), \end{array}$$

(3) $$\begin{array}{c} IG\left({\text{X}}_{i},Y\right)=H\left(Y\right)-H\left(Y\;|\;{\text{X}}_{i}\right), \end{array}$$

where *X*_*i*_ represents the random variable of *i*th feature value, *x* ∈ X_*i*_ denotes the possible value of random variable *X*_*i*_, *p*(*x*) represents the probability when the random variable *X*_*i*_ takes the value *x*, and *Y* denotes the random variable of whether or not a cerebral infarction. *IG*(*X*_*i*_, *Y*) represents the information gain which is used to measure the reduction of uncertainty of event *Y* after *X* is known.

Theoretically, the feature was effective when the value was greater than zero, but we chose 0.1 as the minimal threshold to prevent calculating error and sampling error [18]. That is, features below the threshold were considered to be insignificant. The higher the information gain value of features, the greater contribution to remove noise and retain significant feature information.

### 2.5. Classifier Establishment

The classifier was established to demonstrate the separability of ACI. Given a candidate region, classifier automatically distinguished lesion or normal region in terms of the selected features. The classifiers probably make mistakes, so a classification accuracy score was calculated when all candidate regions were performed, which represents the separability of ACI.

In the experiment, we obtained different classification scores with different features, respectively, which was to confirm the effectiveness of features under different thresholds. We chose 10 common classifiers to obtain a reliable result, calculated the average classification accuracy, and selected the best classification accuracy as the final result. Each classifier experiment was repeated 100 times for average, and 4-fold cross validation was operated to get stable result.

The separation analysis was operated on ACI regions and their symmetrical regions in CT images. In addition, to exclude the separability of the left and right sides of the normal brain, and to explain the separability of the lesion and normal brain at same region, we performed the same experiments on the normal brain and their symmetrical normal regions, as well as normal brain and lesion regions.

## 3. Results

Demographic characteristics of all the patients in this study were shown in Table 1. For each of the 38 patients, one slice from CT was selected, which had a prominent lesion in MRI correspondingly. A total of 38 slices, which are 38 ACI regions and 38 symmetric noninfarct regions, were obtained. We extracted 1301 radiomic features from the candidate regions; meanwhile, the information gain was calculated to extract key information from abundant features. As shown in Table 2, there were 280 features with information gain greater than 0.1, which were considered to be contributory to classify candidate regions. There were 23 features with information gain greater than 0.2, and 2 features with information gain greater than 0.3, which showed potential capability for separating lesion regions and their symmetric noninfarct regions. The related features were used to build up 10 classifiers to verify the feature effectiveness in candidate regions. The average classification accuracy was 0.6467, and the best classification accuracy was 0.7748 (Table 3).

For each of the 18 patients with no positive finding in either CT or MRI, three slices from CT were selected to augment data, which depicted by projecting the lesion labels obtained from 38 aforementioned MRI. A total of 54 slices, which are 54 normal brain tissue regions and 54 symmetrical regions, were obtained. As shown in Table 2, there were only 176 features with information gain greater than 0.1, 1 feature with information gain greater than 0.2, and no feature with information gain greater than 0.3. Although the best classification accuracy was 0.6782, from the overall classification results, the classification results were generally low and the average classification accuracy was only 0.5414 (Table 3).

For each of the 56 aforementioned patients, one slice from CT were selected. A total of 56 slices, which are 38 lesion regions and 18 normal brain tissue regions, were obtained. As shown in Table 2, there were 501 features with information gain greater than 0.1, 126 features with information gain greater than 0.2, 51 features with information gain greater than 0.3, 18 features with information gain greater than 0.4, and 1 feature with information gain greater than 0.5. The average classification accuracy was 0.7480, and the best classification accuracy was as high as 0.8694 (Table 3).

**Figure 5:**
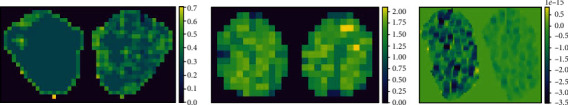
Feature map of (a) lesion region (left) and its symmetric region (right) showed separable by calculating short-run low gray-level emphasis on the square transformed images, (b) normal region (left) and its symmetric region (right) showed inseparable by calculating run entropy on the wavelet transformed images, and (c) lesion region (left) and same position of normal region (right) showed separable by calculating 10th percentile on the wavelet transformed images.

Besides, feature map that features reflected on candidate regions were shown to illustrate the effectiveness of feature analysis (Figure 5). We visualized one of the first three features ordered by information gain on the candidate region. Among them, it is a clear distinction on lesion and its symmetrical region, which explains the separability of ACI. The left and right sides of the normal region showed no obvious difference, which confirmed the inseparability of the left and right sides of the normal brain. The difference between lesion and normal region was also prominent, which indicated separability of lesion and normal region.

## 4. Discussion

Sensitively recognizing acute cerebral infarction is a valuable research for clinical treatment, within effective thrombolytic time. To the best of our knowledge, the finding of analyzing the separability of acute cerebral infarction lesions in CT based on image registration, precision positioning, radiomic feature extraction, and information gain calculation has not previously been well established in the literature. The overall concept of the algorithm was to extract and analyze the feature of regions where there is cerebral infarction, and more importantly, to separate lesions from normal regions.

Accurately recognizing acute ischemic stroke by CT remains challenging, due to the low accuracy of radiologist diagnosis. A lot of studies focused on prior knowledge, including decrease of CT attenuation, loss of gray-white matter differentiation, and sulcal effacement. However, 1/3 cases were missed since the sensitivity and specificity were low [5]. On the other hand, acute ischemic stroke is inconspicuous, more complex features including texture, need to be introduced [19, 20]. Later, Rajini [11] proposed a method to separate ischemic stroke regions from normal tissues in CT, which used segmentation, midline offset, and image features. Nevertheless, most of these studies involved acute lesions that were already visible. Instead, Chawla [12] investigated a two-stage classification system by comparing the image intensity differences between the two hemispheres, which can detect hemorrhagic and ischemic stroke. Recently, a predictive model based on Ranklet features to distinguish strokes and normal tissues was proposed, which achieved significantly high accuracy 81% [13]. Compared with MRI, it is still a gap which is not sufficient for practice, and artificial intelligence-assisted CT diagnosis needs more robust features. As Petrou [21] suggested, a few features, which are not sensitive to human vision and tend to be ignored, are needed to be excavated. Therefore, it is potential for improving the accuracy of detection ACI by exploring other features besides texture.

Lesion delineation is the primary premise in medical image analysis. However, defining the entire lesion boundaries in CT might be complicated because of the invisible of lesion. The next practical way is that ischemic tissues can be highlighted by comparing the left and right sides, since inherent anatomical structures in the human brain are symmetric [22, 23]. Nevertheless, it is unreliable by simply comparing both sides of the brain especially the lesion size was small because of normal brain tissue overwhelming the characteristic features from small lesion.

In order to solve the conundrum of candidate region acquisition and feature quantity insufficiency, we matched exact lesion regions from DWI to CT images. The exact lesion regions and their symmetrical regions served as candidate regions for imaging feature extraction. Inspired by Lambin et al. [14] who extracted a large number of radiomic features from medical images and used statistical analysis to identify features that could characterize disease, we extracted 1301 radiomic features through image transformation and feature extraction. Note that we do not claim any novelty in the extraction design. Instead, our contribution lies in the essence of that constructing more complex feature is necessary for selecting certain features which contribute to classification in the next step. Since not all features were effective, the information gain was further introduced as a standard to measure the correlation between features and ACI, which is according to the principle of feature distinction and independence in mathematical description [16–18]. Furthermore, machine learning is often used as a means to evaluate radiomic analysis [24–26]. Hence, 10 classifiers were established with selected features to verify the effectiveness.

The sufficient experimental data showed differences between the cerebral infarction and their symmetrical noninfarct regions, since the effective features extracted had great potential in classify lesions and their symmetrical regions. Simultaneously, to rule out these separable differences probably coming from the inherently separable between the left and right sides of the normal brain, we operated on normal brain tissue and their symmetrical regions. The results confirmed that the left and right sides of the normal brain tissues were inseparable. According to effective features achieved astounding performance in classify lesion regions and same position of another normal brain tissue, the lesions which were separable from normal tissue in CT were further confirmed. More importantly, the classification results proved the necessity and effectiveness of feature extraction and screening.

Some limitations are noteworthy in the current study. We only included 18 patients performed head CT with no positive finding. They were younger compared to 38 patients performed with both CT and MRI and diagnosed as ACI by DWI, since it is difficult to select normal brain tissue in the elderly. Besides, the size of our population might be considered small; further studies that include a larger population are needed to strengthen the statistical power of these investigations.

## 5. Conclusion

This study analyzed the separability of acute cerebral infarction lesions in CT, which facilitates CT diagnosis directly. Furthermore, the results of the study established a theoretical foundation for artificial intelligence-assisted CT diagnosis, which will bring potential benefits for acute cerebral infarction patients: shortening the waiting time of thrombolysis, saving the cost of examination, and improving the prognosis.

## Figures and Tables

**Table 1:** Demographic characteristic and multivariate logistic regression results.

Characteristic	Total (*n* = 56)	Patients with ACI (*n* = 38)	Patients with no ACI (*n* = 18)	OR^*α*#^ (OR 95% CI)
Age∗		64.71 ± 12.92	34.17 ± 6.52	1.458 (1.086~1.957)
Sex(y)†				
Woman	24	17 (44.74)	7 (38.89)	1.000
Man	32	21 (55.26)	11 (61.11)	2.748 (0.108~69.973)
Predict value				
Negative	10	4 (10.53)	6 (33.33)	1.000
Positive	46	34 (89.47)	12 (66.67)	43.530 (0.640~2960.497)
MRI		Diagnosed as ACI	No positive finding	
CT		No positive finding	No positive finding	

^#^The value of OR was obtained from binary logistic regression by adjusting *α*_in_ = 0.1, and *α*_out_ = 0.15. Dependent is the true value, and covariates are sex, age, and the predicted value. All the covariates were calculated by the enter method. ^∗^Data are mean ± standarddeviation. ^†^Data in parentheses are percentages. ACI denotes acute cerebral infarction.

**Table 2:** Feature number under different thresholds of information gain on candidate region.

Candidate region	Threshold					
	0.0	0.1	0.2	0.3	0.4	0.5
Feature number of lesions and their symmetrical regions	1292	280	23	2	0	0
Feature number of normal and their symmetrical regions	1279	176	1	0	0	0
Feature number of lesions and normal regions	1295	501	126	51	18	1

**Table 3:** The classification accuracy result with selected features under different thresholds of information gain on candidate region.

Classifier	Lesions and their symmetrical regions threshold				Normal and their symmetrical regions threshold			Lesions and normal regions threshold					
	0.0	0.1	0.2	0.3	0.0	0.1	0.2	0.0	0.1	0.2	0.3	0.4	0.5
Multilayer perceptron	0.4902	0.4974	0.5694	0.7269	0.5061	0.4983	0.4434	0.5476	0.5242	0.5291	0.5292	0.5125	0.7185
Decision tree	0.5980	0.6138	0.6603	0.6655	0.5941	0.6333	0.5841	0.7155	0.7423	0.7782	0.7742	0.7982	0.8230
Random forest	0.5897	0.6452	0.7036	0.7206	0.6055	0.6782	0.5775	0.7700	0.7932	0.8401	0.8260	0.8162	0.8360
Adaboost	0.5850	0.6263	0.6811	0.6818	0.5946	0.6651	0.6001	0.7071	0.7276	0.7671	0.7862	0.7748	0.8291
Gradient boosting	0.5977	0.6346	0.6838	0.7463	0.5931	0.6505	0.5950	0.7517	0.7564	0.7903	0.7978	0.8017	0.8694
Bagging	0.6217	0.6567	0.6973	0.7249	0.6065	0.6529	0.5745	0.7530	0.7767	0.8169	0.8282	0.8144	0.8307
Bernoulli naive Bayes	0.5100	0.6164	0.6724	0.7105	0.4413	0.5175	0.4318	0.6557	0.6748	0.7253	0.7594	0.7566	0.6785
Gaussian naive Bayes	0.4743	0.6203	0.6661	0.6984	0.4801	0.4574	0.4439	0.3737	0.3935	0.8098	0.7842	0.8323	0.8605
Support vector machine	0.4184	0.4223	0.6903	0.4211	0.4382	0.4299	0.4326	0.6785	0.6785	0.6650	0.8123	0.7942	0.6785
*K*-nearest neighbor	0.2686	0.4563	0.7188	0.7748	0.2690	0.3492	0.6137	0.5812	0.5585	0.6437	0.8153	0.8010	0.8673

Average		0.5789	0.6743	0.6870		0.5532	0.5296		0.6625	0.7365	0.7712	0.7701	0.7991
			0.6467			0.5414					0.7480		

## Data Availability

The data used to support the findings of this study is available from the corresponding author upon request.

## Abbreviations

ACI: Acute cerebral infarction
DWI: Diffusion-weighted imaging.
